# EpCAM^high^ and EpCAM^low^ circulating tumor cells in metastatic prostate and breast cancer patients

**DOI:** 10.18632/oncotarget.26298

**Published:** 2018-11-02

**Authors:** Sanne de Wit, Mariangela Manicone, Elisabetta Rossi, Rita Lampignano, Liwen Yang, Beate Zill, Alvera Rengel-Puertas, Marianne Ouhlen, Mateus Crespo, Anne Margreet Sofie Berghuis, Kiki Carlijn Andree, Riccardo Vidotto, Elisabeth Katharina Trapp, Marie Tzschaschel, Emeline Colomba, Gemma Fowler, Penelope Flohr, Pasquale Rescigno, Mariane Sousa Fontes, Rita Zamarchi, Tanja Fehm, Hans Neubauer, Brigitte Rack, Marianna Alunni-Fabbroni, Françoise Farace, Johann De Bono, Maarten Joost IJzerman, Leonardus Wendelinus Mathias Marie Terstappen

**Affiliations:** ^1^ Department of Medical Cell BioPhysics, Faculty of Sciences and Technology, MIRA Institute, University of Twente, Enschede, The Netherlands; ^2^ Veneto Institute of Oncology IOV–IRCCS, Padova, Italy; ^3^ DiSCOG–University of Padova, Padova, Italy; ^4^ Department of Obstetrics and Gynecology, Heinrich-Heine-University, Düsseldorf, Germany; ^5^ Department of Gynecology and Obstetrics, Ludwig-Maximilians-University, Munich, Germany; ^6^ “Circulating Tumor Cells” Translational Platform, Gustave Roussy, Université Paris-Saclay, Villejuif, France; ^7^ “Identification of Molecular Predictors and New Targets for Cancer Treatment”, Gustave Roussy, Université Paris-Saclay, Villejuif, France; ^8^ Cancer Biomarkers, The Institute of Cancer Research, Sutton, United Kingdom; ^9^ Department of Health Technology and Services Research, Faculty of Behavioural, Management and Social Sciences, MIRA Institute, University of Twente, Enschede, The Netherlands; ^10^ Department of Gynecology and Obstetrics, Ulm-University, Munich, Germany; ^11^ Department of Medicine, Gustave Roussy, Université Paris-Saclay, Villejuif, France; ^12^ Prostate Cancer Targeted Therapy Group, The Royal Marsden NHS Foundation Trust, The Institute of Cancer Research, Sutton, United Kingdom

**Keywords:** circulating tumor cells (CTC), EpCAM, castrate-resistant prostate cancer (CRPC), metastatic breast cancer (mBC), epithelial-to-mesenchymal transition (EMT)

## Abstract

The presence of high expressing epithelial cell adhesion molecule (EpCAM^high^) circulating tumor cells (CTC) enumerated by CellSearch^®^ in blood of cancer patients is strongly associated with poor prognosis. This raises the question about the presence and relation with clinical outcome of low EpCAM expressing CTC (EpCAM^low^ CTC). In the EU-FP7 CTC-Trap program, we investigated the presence of EpCAM^high^ and EpCAM^low^ CTC using CellSearch, followed by microfiltration of the EpCAM^high^ CTC depleted blood. Blood samples of 108 castration-resistant prostate cancer patients and 22 metastatic breast cancer patients were processed at six participating sites, using protocols and tools developed in the CTC-Trap program. Of the prostate cancer patients, 53% had ≥5 EpCAM^high^ CTC and 28% had ≥5 EpCAM^low^ CTC. For breast cancer patients, 32% had ≥5 EpCAM^high^ CTC and 36% had ≥5 EpCAM^low^ CTC. 70% of prostate cancer patients and 64% of breast cancer patients had in total ≥5 EpCAM^high^ and/or EpCAM^low^ CTC, increasing the number of patients in whom CTC are detected. Castration-resistant prostate cancer patients with ≥5 EpCAM^high^ CTC had shorter overall survival versus those with <5 EpCAM^high^ CTC (*p* = 0.000). However, presence of EpCAM^low^ CTC had no relation with overall survival. This emphasizes the importance to demonstrate the relation with clinical outcome when presence of CTC identified with different technologies are reported, as different CTC subpopulations can have different relations with clinical outcome.

## INTRODUCTION

The presence of circulating tumor cells (CTC) expressing the cell surface epithelial cell adhesion molecule (EpCAM) as well as intracellular cytokeratins (CK), are associated with poor outcome in patients with metastatic as well as non-metastatic disease [1–8]. In the CellSearch^®^ system, CTC that show no or low expression of EpCAM are discarded during magnetic isolation and their information is lost. This raises the question how many of these EpCAM-negative or EpCAM low expressing CTC (hereinafter referred to as EpCAM^low^ CTC) are present and whether or not their presence is also associated with poor outcome. In the FP7 EU program CTC-Trap (https://www.utwente.nl/tnw/ctctrap/) this question was investigated by collecting the blood discarded by the CellSearch system after immunomagnetic enrichment of EpCAM^high^ cells. This sample is passed through a microfilter and cells remaining on the filter were fluorescently labeled for EpCAM^low^ CTC scoring [9]. To validate this method for detection of epithelial EpCAM^low^ CTC, the procedure was tested at six different sites in the CTC-Trap consortium. Healthy donor blood spiked with cells of the prostate cancer cell line PC3 (1.0 × 10^4^ EpCAM antigens, average size 17.7 μm) and the breast cancer cell line MDA-MB-231 (1.5 × 10^4^ EpCAM antigens, average size 15.6 μm) were tested for recovery of EpCAM^high^ and EpCAM^low^ cells. Subsequently, the presence of EpCAM^high^ and EpCAM^low^ CTC was investigated in 108 CRPC patients and 22 metastatic breast and their presence was related to overall survival.

## RESULTS

### Validation of CTC detection protocols across sites

**Figure 1:**
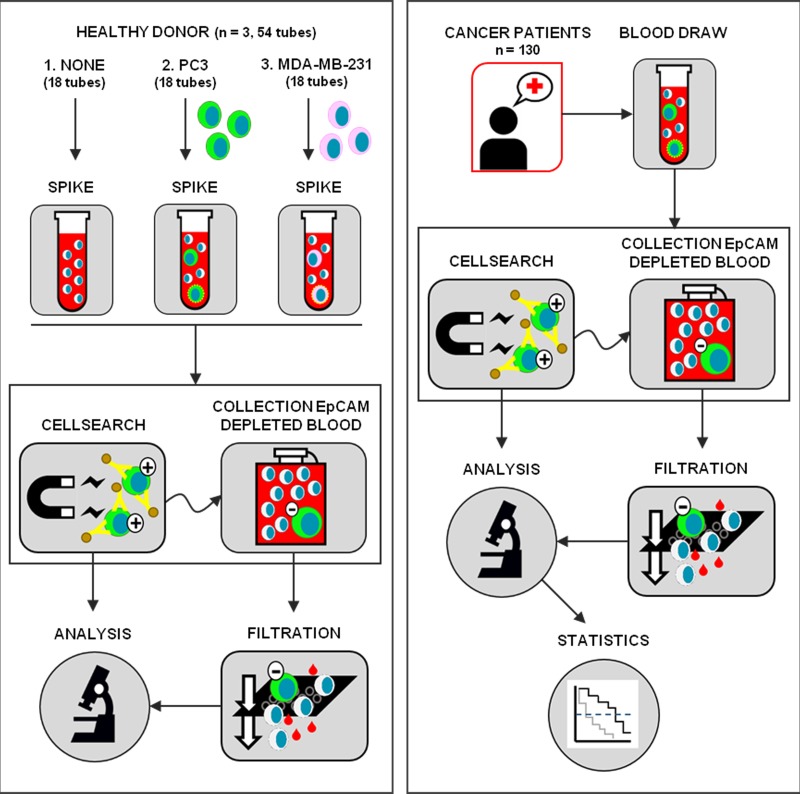
Schematic representation of the study Left panel: one site collected blood from three healthy donors and prepared three tubes for each of the six sites (*Spike*) to send for enumeration of EpCAM^high^ cell line CTC (*CellSearch*) and EpCAM^low^ cell line CTC (*Collection EpCAM Depleted Blood* and *Filtration*): no cells were spiked in tube 1, tube 2 was spiked with on average 270 PC3 cells and tube 3 was spiked with on average 270 MDA-MB-231 cells. This process was repeated three times with three different healthy donors. Right panel: blood samples collected from 108 castrate-resistant prostate cancer patients and 22 metastatic breast cancer patients (*Blood Draw*) at each clinical site were processed for enumeration of EpCAM^high^ CTC (*CellSearch*), followed by detection of EpCAM^low^ CTC (*Collection EpCAM Depleted Blood* and *Filtration*). CTC are detected by fluorescence microscopy and scored (*Analysis*) and the results are related to outcome (*Statistics*).

**Figure 2:**
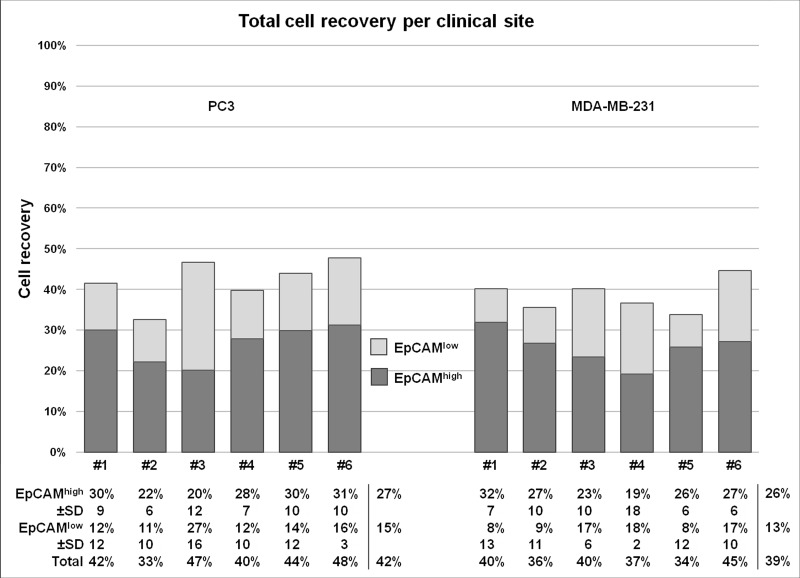
Recovery of PC3 and MDA-MB-231 cancer cells for each clinical site Recovery of on average 270 PC3 cells and MDA-MB-231 cells spiked in each blood sample, processed with the methods for EpCAM^high^ and EpCAM^low^ CTC detection. The samples were processed with CellSearch to determine the recovery of EpCAM^high^ CTC and the EpCAM depleted blood was filtered through 5 μm microsieves to determine the recovery of EpCAM^low^ cells. In total, per site (labeled #1–6) three samples per cell line and three negative controls were processed. The average recovery per three samples is displayed with its standard deviation, followed by an average recovery of all sites per method and cell line.

On average, 270 PC3 or MDA-MB-231 cells were spiked per tube in 36 7.5 mL blood samples of three healthy donors and sent to the six sites for processing according to the established protocols (Figure 1). The recovery of PC3 (median size 17.7 μm) and MDA-MB-231 cells (median size 15.6 μm) was determined using CellSearch (for EpCAM^high^ cells) and on microsieves (for EpCAM^low^ cells), after filtration of the blood discarded by CellSearch. The distribution of the EpCAM density is illustrated in Supplementary Figure 1. The results are illustrated in Figure 2 and a detailed overview of cell recovery per site is provided in Supplementary Figure 2. For EpCAM^high^ CTC detected by CellSearch, the recovery of PC3 cells varied between 11.9% and 40.6% (mean 26.9% ± 9 standard deviation (SD)) and for MDA-MB-231 between 2.7% and 39.0% (mean 25.7% ± 10 SD). For EpCAM^low^ tumor cells detected on the microsieves after filtration of the CellSearch discarded blood, the recovery of PC3 cells varied between 0.6% and 42.5% (mean 15.1% ± 11 SD) and for MDA-MB-231 between 0% and 29.3% (mean 12.8% ± 9 SD). In 18 7.5 mL blood samples obtained from three healthy donors, who donated together all 54 samples, no EpCAM^high^ and no EpCAM^low^ cells were detected.

### EpCAM^high^ and EpCAM^low^ CTC in blood from metastatic breast and prostate cancer patients

**Figure 3:**
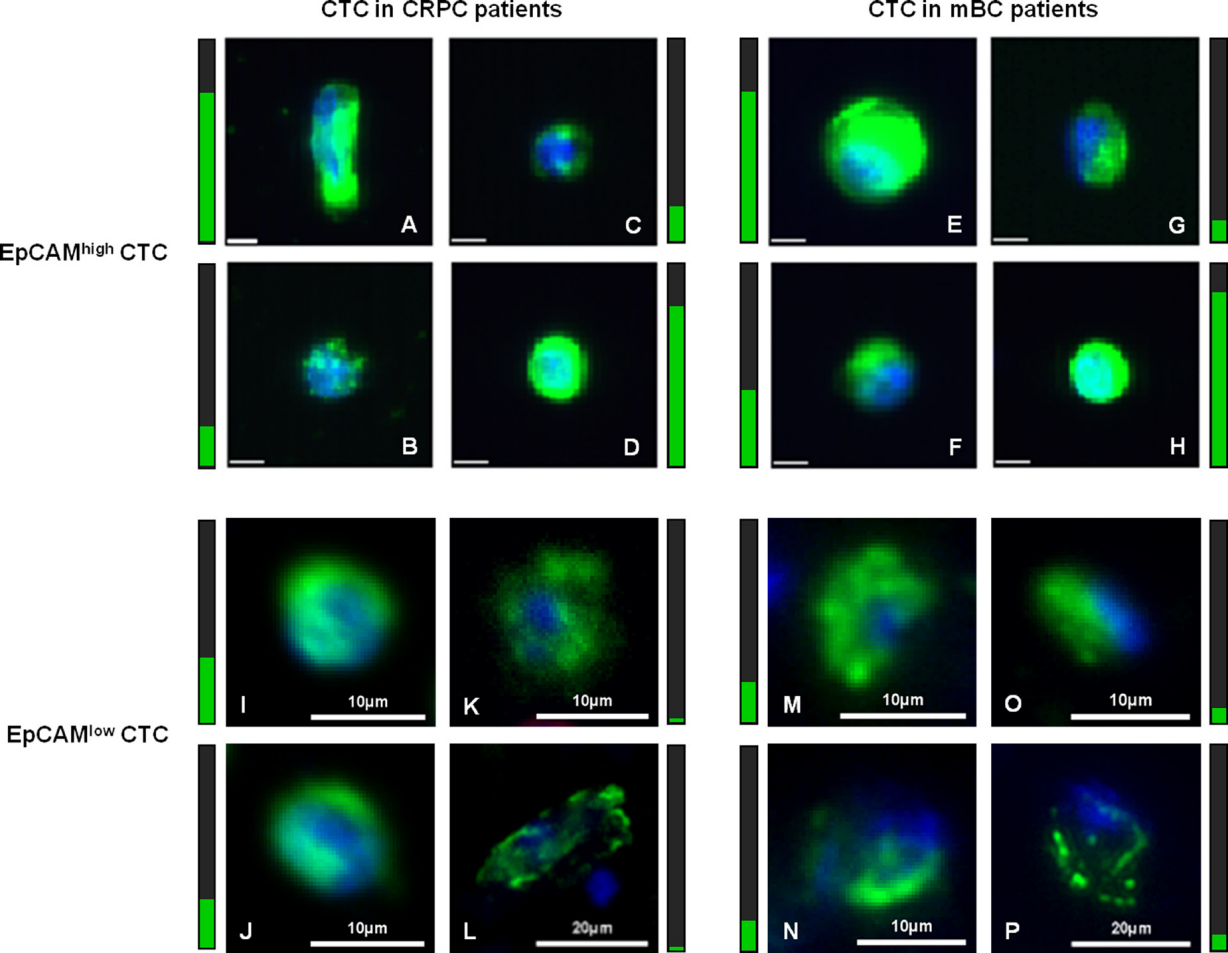
Gallery of captured CTC in CRPC and mBC patients Display of EpCAMhigh CTC (panels **A**–**H**) captured with CellSearch and EpCAMlow CTC (panels **I**–**P**) captured with microsieves in castrate-resistant prostate cancer patients (left panels **A**–**D** and **I**–**L**) and metastatic breast cancer patients (right panels **E**–**H** and **M**–**P**). Cells in panel (**A**–**B**) are from the same patient, as are the cells in panels (**C**–**D**, **E**–**F**, **G**–**H** and **I**–**J**). The value for intensity of CK staining (green) is represented in the vertical bar next to the image, visualizing a very high intensity value with maximum 4095 counts as a full green bar. The nucleus is stained with DAPI (blue). The unlabelled scale bar for EpCAM^high^ CTC images is 6.4 μm.

EpCAM^high^ and EpCAM^low^ CTC were enumerated in 7.5 mL blood obtained from 108 CRPC and 22 mBC patients. A detailed overview of the number of CTC detected in the patients is provided in Table 1, whereas the actual CTC numbers for each patient is listed in Supplementary Table 1 and visualized in Supplementary Figure 3. In CRPC patients, EpCAM^high^ CTC ranged from 0–3300 (median: 6, mean: 124 ± 400 SD) and EpCAM^low^ CTC ranged from 0–24 (median: 1, mean: 3 ± 4 SD). In mBC patients, EpCAM^high^ CTC ranged from 0–208 (median: 1, mean: 13 ± 43 SD) and EpCAM^low^ CTC ranged from 0–35 (median: 3, mean: 3 ± 11 SD). In CRPC patients, ≥5 EpCAM^high^ CTC or ≥5 EpCAM^low^ CTC were detected in 56 (53%) and 26 (28%) patients, respectively. In the group of mBC patients, 7 (32%) showed ≥5 EpCAM^high^ CTC and 8 (36%) ≥5 EpCAM^low^ CTC. Summing EpCAM^high^ CTC and EpCAM^low^ CTC to ≥5 CTC, we observed in 70% positive CRPC (*n* = 91) and 64% positive mBC patients, increasing the combined CTC-positivity rates by 32% and 100% respectively, in comparison to positivity rates for EpCAM^high^ CTC only. In total, 37% CRPC patients and 23% mBC patients had ≥5 EpCAM^high^ CTC, but <5 EpCAM^low^ CTC. Vice versa, <5 EpCAM^high^ CTC and ≥5 EpCAM^low^ CTC were detected in 10% CRPC and in 18% mBC patients. Figure 3 presents a gallery of EpCAM^high^ CTC (upper panels 3A–3H) and EpCAM^low^ CTC (lower panels 3I–3P) that were found in CRPC patients (left panels 3A–3D and 3I–3L) and mBC patients (right panels 3E–3H and 3M–3P), showing CTC of various sizes and staining of CK intensity.

**Table 1:** Frequency of CTC in CRPC and mBC patients

Prostate cancer							Breast cancer				
% patients with EpCAM^high^ CTC							% patients with EpCAM^high^ CTC				
	# CTC	0	1–4	≥5	≥10	Total	0	1–4	≥5	≥10	Total
**% patients with EpCAM^low^ CTC**	**0**	12.1	9.9	22.0	18.7	*44.0*	18.2	4.5	9.1	9.1	*31.8*
	**1–4**	5.5	6.6	15.4	14.3	*27.5*	9.1	9.1	13.6	4.5	*31.8*
	**≥5**	7.7	2.2	18.7	16.5	*28.6*	13.6	13.6	9.1	4.5	*36.3*
	**≥10**	1.1	0	5.5	4.1	*6.6*	9.1	9.1	9.1	4.5	*27.3*
	**Total**	*25.3*	*18.7*	*56*	*49.5*	*100*	*40.9*	*27.3*	*31.8*	*18.2*	*100*

The frequency (%) of EpCAM^high^ and EpCAM^low^ CTC in 7.5 mL of blood of 91 castration-resistant prostate cancer patients and 22 metastatic breast cancer patients.

### Overall survival of CRPC patients with EpCAM^high^ and EpCAM^low^ CTC

Follow-up data from patients that were enrolled in ongoing clinical trials could not be obtained at the time this manuscript was written. Therefore, follow-up data could only be obtained from 85 out of 108 CRPC patients and 16 out of 22 mBC patients. The cohort of mBC patients that remained was too small for survival analysis and was therefore omitted.

**Figure 4:**
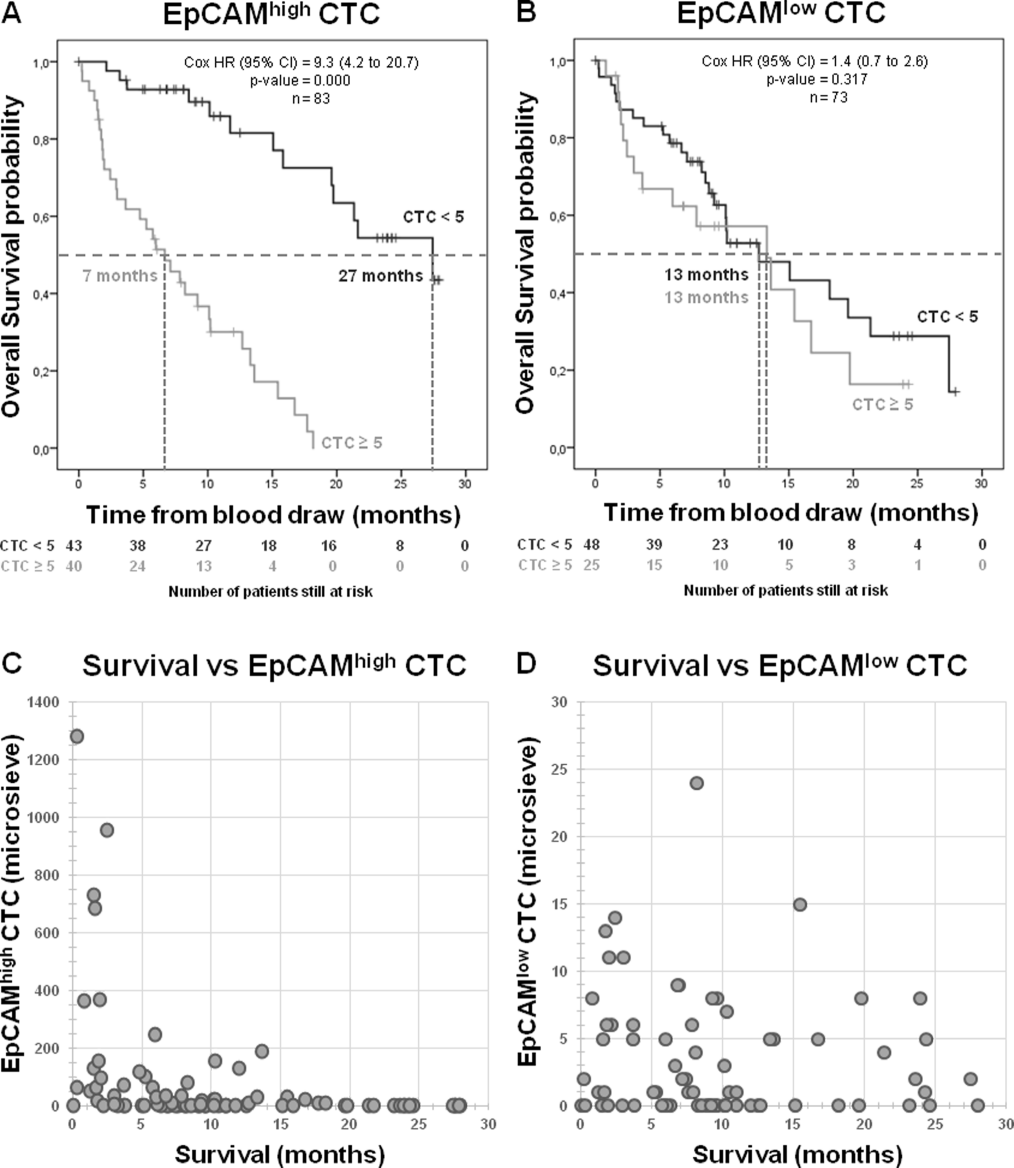
Overall survival for CRPC patients Kaplan–Meier curve of overall survival of patients with EpCAM^high^ CTC from CellSearch (**A**), EpCAM^low^ CTC from microsieves (**B**) show a strong correlation between EpCAM^high^ CTC and survival, but no correlation between EpCAM^low^ CTC and survival. Scatter plot of survival versus the amount of EpCAM^high^ CTC (*n* = 83) visualizes the expected trend that most patients with high number of CTC have a short survival (**C**), whereas the amount EpCAM^low^ CTC in patients (*n* = 73) show no such trend (**D**).

To relate the presence of CTC in CRPC patients to overall survival, the patient cohort was split into a favorable group and an unfavorable group, using 5 CTC as a threshold [2, 4]. In Figure 4 the Kaplan-Meier curves for EpCAM^high^ and EpCAM^low^ CTC are shown. A significant difference is observed for the presence of ≥5 EpCAM^high^ CTC in relation to overall survival (*p* = 0.000) (Figure 4A), whereas no significant difference is observed for ≥5 EpCAM^low^ CTC (*p* = 0.317) (Figure 4B). The combination of EpCAM^high^ CTC and EpCAM^low^ CTC was related with overall survival by separating the cohort into four groups (Supplementary Figure 4A). This shows again that the strong correlation with survival can be solely contributed to EpCAM^high^ CTC and not to EpCAM^low^ CTC (*p* = 0.000). Since ≥5 EpCAM^low^ CTC show no correlation with survival, perhaps a lower CTC cut-off value would show a correlation. However, the scatter plot of the number of EpCAM^low^ CTC versus survival of these patients in Figure 4D shows no trend between these two factors, whereas this trend is visible between survival and EpCAM^high^ CTC (Figure 4C). A receiver operating characteristic (ROC) curve was then used to determine the highest diagnostic cut-off value for EpCAM^low^ CTC. Although this calculates a threshold at ≥1 EpCAM^low^ CTC, this value can be considered inconclusive since the separation between sensitivity and specificity is very low (Supplementary Figure 4B). Using the threshold of ≥1 EpCAM^low^ CTC also no relation can be observed with overall survival (*p* = 0.748) (Supplementary Figure 4C).

## DISCUSSION

In this multicenter study we determined the presence of EpCAM^high^ and EpCAM^low^ CTC in castration resistant prostate cancer and metastatic breast cancer patients. Protocols and tools for detection were developed in the FP7-program CTC-Trap and validated at six clinical sites cooperating in the program. The current standard CellSearch method for CTC enumeration was used for the detection of CTC expressing EpCAM, followed by capturing and filtering of the sample depleted of these EpCAM^high^ CTC, and stained for detection of epithelial CTC expressing no or low EpCAM (Figure 1).

To validate the procedure of CellSearch followed by filtration for detection of EpCAM^low^ CTC, samples spiked with cells from the PC3 or MDA-MB-231 cancer cell lines were used. In the EpCAM^high^ CellSearch fraction, 71% of the PC3 total spiked cells were recovered and 74% of the spiked MDA-MB-231 cells. The actual expression levels of EpCAM on EpCAM expressing CTC is an important question. Rao *et al.* reported the EpCAM expression on 100 blood samples of metastatic cancer patients in which CTC were detected by flow cytometry and reported an average EpCAM density of 5.0 × 10^4^ EpCAM antigens [10]. This density is fivefold higher than that of the PC3 (on average 1.0 × 10^4^ EpCAM antigens) and MDA-MB-231 (on average 1.5 × 10^4^ EpCAM antigens) cells used in this study. However, these numbers are the average density and the actual distribution of the EpCAM expression can be quite broad, as illustrated in Supplementary Figure 1 [10]. The data supports our notion that the majority of CTC with relatively high EpCAM antigen density are captured by CellSearch, while CTC expressing no EpCAM or low EpCAM levels will be present in the EpCAM depleted fraction. After passage of the EpCAM depleted blood through microsieves, only a portion of the spiked cells –and most likely also CTC – will be captured.

Relative recovery of PC3 cells and MDA-MB-231 in the EpCAM^high^ fraction (CellSearch) and EpCAM^low^ fraction (microsieve), as shown in Figure 2, followed the expectations given the size and EpCAM distribution of both cell lines. Although in line with previous reports, the absolute number recovered cells is relatively low, thereby raising the question where and how these cells were lost [9, 11]. Only a small part of cells are destroyed during the CellSearch procedure, indicated by the 90% recovery of cells that express EpCAM at a very high density [9]. Therefore, it is likely to assume most are lost before measurement or during the filtration process. Some of the cells may be missed because they lack sufficient immunostaining for detection, or the cells have simply been degraded before or during the process, as they were not extensively fixed apart from the light fixative used during spiking the blood samples.

The observed recovery rates retrieved for each cell line includes variation introduced by EpCAM density, by different operators at each clinical site and by the three measurements for each cell line (Figure 2 and Supplementary Figure 2). Clearly, the variation in the obtained results vary a lot between the sites, stressing the need for standardization and proficiency testing to assure accurate reporting of actual CTC numbers. Variation introduced by the interpretation of the images of the CTC can be eliminated by the recently introduced ACCEPT open-source imaging program [12, 13].

In both CRPC and mBC patients, EpCAM^high^ and EpCAM^low^ CTC were detected in 7.5 mL blood (Table 1). The number of patients with positive detection of CTC can be increased when both EpCAM^high^ and EpCAM^low^ CTC are used for the ≥5 CTC threshold. This corresponds to our previous report of a small pilot study with 28 non-small cell lung cancer (NSCLC) patients [9]. Also, these findings fit in the trend of recently published filtration based studies, which are reporting large amounts of CTC detected when compared to CellSearch [6, 14–17]. A biological reason for this increase in CTC counts is by many thought to be related to epithelial-to-mesenchymal transition (EMT), which is accompanied by loss of epithelial markers, such as EpCAM and CK, and by an increase of mesenchymal markers, such as vimentin. However, co-expression of epithelial markers and mesenchymal markers on CTC also have been reported [18–25]. In this study, EpCAM^low^ CTC expressing cytokeratins are detected, leaving the question open on the frequency and clinical relevance of EpCAM^low^ CTC lacking CK and expressing mesenchymal markers [21, 22].

To determine whether the presence of EpCAM^high^ and EpCAM^low^ CTC had similar clinical relevance in CRPC patients, we correlated their presence with the time of survival. The threshold of >5 CTC for EpCAM^high^ is the threshold used in the original metastatic breast and prostate studies and was therefore also used for EpCAM^low^ CTC to distinguish between favorable and unfavorable patient groups [3, 23]. Whereas the presence of EpCAM^high^ CTC strongly related to a poor survival (Figure 4A), the presence of EpCAM^low^ CTC did not (Figure 4B). A threshold of ≥1 CTC for EpCAM^low^ CTC also showed no significant relation to survival, as illustrated by the ROC curves and Kaplan-Meier curve presented in Supplementary Figure 4. Similar observations were made in NSCLC and this prompts the question what type of cells these EpCAM^low^ cells truly are [9]. Here, we define these cells as having no or low EpCAM expression, based on the effective depletion of cells expressing higher levels of EpCAM by the CellSearch system and relatively low capture of cells expressing low levels of EpCAM (Figure 2). The EpCAM^low^ cells have a nucleus identified by DAPI, express CK, but not CD45. The presence of CK shows that these cells are of epithelial origin, but do not proof that they are indeed tumor cells [24]. The CK expression in CTC is variable, even in the same patient (Figure 3). EpCAM^low^ cells that resemble the morphology of EpCAM^high^ CTC (Figure 3A–3H) have a higher intensity and filamentous pattern of CK (Figure 3I, 3J), whereas low CK intensity cells show irregular CK expression (Figure 3K–3P). This may indicate that these cells are different subtypes of CTC, where each subpopulation could express its own characteristics and clinical behavior [25]. Even so, the intensity of CK staining can be very low in some patients making it difficult for operators to score CTC. Even using clearly defined criteria for operators to follow in scoring CTC, the discrepancy between operators can be high and argues for an automated approach to analyze images for cells of interest [12, 13]. Definitive proof of the cancerous nature of the EpCAM^low^ cells will need to come from the genetic analysis of the cells, as well as the differences between the genetic and proteomic make-up of the EpCAM^high^ and EpCAM^low^ cells.

Concluding, in this multicenter study we validated the detection of EpCAM^high^ and EpCAM^low^ CTC from the same blood draw tube using the CellSearch system for enumeration of the EpCAM^high^ and by microscopic examination of EpCAM^low^ after passage of the blood depleted from EpCAM^high^ cells through microsieves. We showed that the number of CTC detected in both metastatic breast and prostate cancer can be increased when considering both EpCAM^high^ and EpCAM^low^ CTC, but also show that only the presence of EpCAM^high^ CTC, and not EpCAM^low^ CTC, are strongly correlated with poor overall survival. This emphasizes the importance to demonstrate the relation with clinical outcome when presence of CTC identified with different technologies are reported, as different CTC subpopulations can have different relations with clinical outcome.

## METHODS

### Blood samples

Peripheral blood samples were drawn by venepuncture into 10 mL CellSave Preservative Tubes (Menarini Silicon Biosystems, Huntingdon Valley PA, USA) from healthy donors, metastatic breast and castration resistant prostate cancer patients. All patients and healthy donors provided written informed consent and the study protocol was approved by the medical ethical committee's at each participating site. Patient demographics of patients with known clinical outcome are provided in Table 2. The sites in the CTC-Trap consortium collaborating in this validation study are: The Institute of Cancer Research, The Royal Marsden Hospital, NHS Foundation Trust (ICR), United Kingdom; Institute Gustave Roussy (IGR), France, Instituto Oncologico Veneto (IOV), Italy; Ludwig-Maximilians-University Muenchen (LMU), Germany; Universitätsklinikum Düsseldorf (UKD), Germany; and the Universiteit Twente (UT), the Netherlands. CRPC patients (at sites ICR, IGR and IOV) and metastatic breast cancer patients (at sites LMU and UKD) were recruited and processed on site within 96 hours. A schematic overview of the study is shown in Figure 1.

**Table 2:** Patient demographics

Castration-resistant prostate cancer patients	*n* = 85	Metastatic breast cancer patients	*n* = 16
**Age (years)**		**Age (years)**	
Median (range)	71 (49–84)	Median (range)	63 (37–89)
Unknown	24 (28%)	Unknown	1 (6%)
**Status at last follow-up**		**Status at last follow-up**	
Alive	38 (45%)	Alive	12 (75%)
Dead	47 (55%)	Dead	4 (25%)
**Mean follow-up time in months (min-max)**		**Mean follow-up time in months (min-max)**	
Alive	13 (0–28)	Alive	11 (3–17)
Dead	09 (0–27)	Dead	4 (2–8)
**Line of therapy**		**Line of therapy**	
Before therapy	08 (9%)	Before therapy	5 (31%)
1st line	10 (12%)	1st line	2 (13%)
2nd line	19 (22%)	2nd line	1 (6%)
≥3rd line	24 (28%)	≥ 3rd line	7 (44%)
Unknown	25 (29%)	Unknown	1 (6%)

The demographics of the patients in this study with known clinical outcome.

### Cell lines and spiking

Spiking experiments were performed with cells from the prostate carcinoma cell line PC3 and the breast carcinoma cell line MDA-MB-231. These cell lines were chosen for their low expression of EpCAM, which would allow us to find a fraction in both the EpCAM^high^ and EpCAM^low^ fraction. The median size of the MDA-MB-231 cells was 15.6 μm with an average EpCAM density of 1.5 × 10^4^ (165 %CV) antigens. The median size of the PC3 cells was 17.7 μm with an average EpCAM density of 1.0 × 10^4^ (89 %CV) antigens. The histograms showing the distribution of the EpCAM density of the PC3 and MDA-MB-231 cells is shown in Supplementary Figure 1. For comparison, the EpCAM density of the breast cancer cell line MCF-7, expressing EpCAM at much higher density, is shown in the figure as well. All cell lines were obtained from ATCC (Manassa VA, USA) and have not been authenticated in the past four years. They were grown at 37°C and 5% CO_2_. The PC3 cell line was cultured in RPMI-1640 (Life Technologies Corporation, Carlsbad CA, USA), and the MDA-MB-231 cell line was cultured in DMEM (Euroclone, Pero MI, Italy); both media were supplemented with 10% fetal bovine serum (GIBCO, Life Technologies Corporation), 2 mM UltraGlutamine (Lonza, Basel, Switzerland) and 10 mM Hepes (Lonza). The median cell size from both cell lines was determined with a Coulter counter pipette (Scepter, Millipore, Billerica, MA, USA). The EpCAM density was determined using a flow cytometer (FACS ARIA II, BD Biosciences, San Jose, CA, USA) and QuantiBrite beads (BD Biosciences).

The MDA-MB-231 cells and PC3 cells were spiked in healthy donor CellSave blood. Cell numbers for spiking were counted manually with a fluorescent microscope, using the nuclear dye Hoechst 33342 for visualization on a glass slide. Targeting a spike number of 250 cells in 7.5 mL blood volume, the exact number of cells was counted before adding the cells to the blood and subsequently used to determine the recovery in the CellSearch and on the microsieve. On average, 270 cells were spiked per tube. Unspiked blood samples from healthy volunteers were used as a negative control. Spiked samples were prepared at the IOV laboratory and distributed under temperature control to the six sites to be processed after 48 hours. Each site received a tube of unspiked blood (labeled A), one tube spiked with PC3 cells (labeled B) and one tube spiked with MDA-MB231 cells (labeled C), all from one donor. This was repeated three times with three different donors.

### CTC detection by CellSearch

CTC were enumerated in aliquots of 7.5 mL of blood with the CellSearch^®^ Circulating Tumor Cell Kit (Menarini Silicon Biosystems). Analysis was performed within 96 hours of the blood draw. Blood samples were enriched for EpCAM^high^ cells and stained with DAPI, Cytokeratin-PE and CD45-APC on the CellTracks Autoprep. Image acquisition of the stained cartridges was performed on the CellTracks Analyzer II. Images of CTC candidates were identified by the CellTracks Analyzer II and presented to experienced operators for classification. Candidates were assigned as CTC when the objects were larger than 4 μm, stained with DAPI and CK, lacked CD45 staining and had morphological features consistent with that of a cell [1].

### Blood sample collection after EpCAM^high^ CTC enrichment

After immunomagnetic selection of EpCAM^high^ cells, the CellTracks Autoprep aspirates the blood that is void of the selected cells and transports it to a waste container outside the instrument. To enable the investigation of this blood for residual tumor cells, the sample was collected manually or with the specific designed Automated Sample Collection Device (ASCD), as described previously [9]. For manual collection of the Autoprep discarded blood sample, the top of the waste container was removed. After visual inspection of presence of blood in the tubing coming out of the Autoprep, a 50 mL conical tube is placed under the outlet and the sample is collected until no blood can be observed anymore in the tubing. This is repeated for each sample. The first blood arrives at the waste container approximately 1 hour after start of the Autoprep. The protocols and tools are described in more detail at https://www.utwente.nl/tnw/mcbp/protocolsandtools/.

### Filtration of the discarded CellTracks Autoprep blood sample

To filter tumor cells from the EpCAM^high^ CTC depleted blood sample, microsieve membranes were used (VyCAP, Deventer, The Netherlands). Each microsieve contains 111,800 pores of 5 μm in diameter and these pores are spaced 14 μm apart in lanes with a porosity of 10% on a total surface area of 8 by 8 mm^2^. The specifications of the microsieves were obtained from previous experiments [9, 11, 26]. The microsieve is contained in a plastic holder that was placed in a disposable filtration unit, which can be placed on a pump unit that maintained a pressure of −100 mbar across a microsieve during filtration (VyCAP). The CellSearch discarded blood sample was transferred to the filtration unit after which the pump was switched on. The collected blood sample, varying between 25 to 40 mL, was passed through in maximum 10 minutes. After complete filtration of the sample or after 10 minutes, the pump was switched off and if there was any unfiltered sample volume remaining, this was removed with a pipette. Details of the volumes that were not filtered were used to determine how much whole blood volume was processed.

### Staining of cells on microsieves

Conditions for staining on microsieves were optimized to assure uniform staining across the microsieve with a minimum of non-specific binding. After filtration, the microsieve was removed and washed once with a permeabilization buffer containing PBS, 1% bovine serum albumin (Sigma-Aldrich, St. Louis MO, USA) and 0.15% saponin (Sigma-Aldrich). Next, this buffer was placed on the sieve and removed after 15 minutes incubation at room temperature. A cocktail of fluorescently labeled antibodies was used to stain the cells on the sieve for 15 minutes at 37°C. The staining solution consisted of the following monoclonal antibodies: three CK antibody clones targeting CK 4, 5, 6, 8, 10, 13, 18 (clone C11), CK 1-8 (clone AE3) and CK 10, 14, 15, 16 and 19 (clone AE1), all conjugated to NTb575 (AcZon s.r.l., Bologna, Italy), and one antibody targeting CD45 (clone HI30) labelled with PerCP (Thermo Fisher Scientific, Waltham MA, USA). The CK-pan cocktail was diluted to a final concentration of 3.5 μg/mL and the anti-CD45 antibody was diluted to 4 μg/mL in PBS/1%BSA/0.05% saponin. After removal of the staining cocktail, the microsieve was washed once and then incubated for 5 minutes at room temperature with PBS/1%BSA. Then the sample was fixed using PBS with 1% formaldehyde (Sigma-Aldrich) for 10 minutes at room temperature. Removal of the fluid during each of the staining and washing steps was done by bringing the bottom of the microsieve in contact with an absorbing material using a staining holder (VyCAP). The microsieve was subsequently covered with ProLong^®^ Diamond Antifade Mountant with DAPI (Thermo Fisher Scientific). A custom cut 0.85 × 0.85 cm^2^ glass cover slip (Menzel-Gläser, Saarbrükener, Germany) was placed on both sides of the microsieve for immediate analysis or storage in the freezer at −30°C.

### Detection of cells on microsieves

Microscopes at each site were equipped with a 20× microscope objective with minimal NA0.45 and the same set of filter cubes. The following filters were used: DAPI (DAPI-50LP-A-NQF) with excitation 377/50 nm, dichroic 409 nm LP, emission 409 nm LP; PE (TRITC-B-NQF) with excitation 543/22 nm, dichroic 562 nm LP, emission 593/40 nm and PerCP (FF02-435/40, FF510-Di02 and FF01-676/29 (customized filter cube)) with excitation 435/40 nm, dichroic 510 nm LP, emission 676/29 nm. All cubes were acquired via Nikon (Semrock, Rochester, NY, USA). Images covering the entire 0.64 cm^2^ surface of the microsieves were acquired and stored.

### Scoring of CTC

Analysis of the fluorescent images generated from the CellSearch cartridges were performed according the instructions of the manufacturer. The fluorescent images from the microsieves were analyzed using the open-source software ICY [27]. Operators were asked to annotate every DAPI+/CK+/CD45− event. In case of clogging of the microsieve, CTC counts were extrapolated to the full volume. Images of CTC were analyzed for their intensity in CK, thereby deducting the background value from the intensity value of CK staining ranging from 0 to 4095 counts.

### Statistical analysis

Statistical analysis was performed in R (version 3.3.0) and SPSS (Statistics 24). For survival analysis, patients were divided in two prognostic groups: favorable for less than 5 CTC and unfavorable ≥5 CTC [2, 4]. Kaplan–Meier curves for overall survival were generated and compared using the Log-Rank test. A receiver operating characteristic curve was used to determine the EpCAM^low^ CTC threshold for the highest diagnostic ability. A *p*-value of < 0.05 was considered to indicate a significant difference.

## SUPPLEMENTARY MATERIALS FIGURES AND TABLE





## Abbreviations

CK: cytokeratins
CRPC: castration-resistant prostate cancer patients
CTC: circulating tumor cells
CV: coefficient of variation
EMT: epithelial to mesenchymal transition
EpCAM: epithelial cell adhesion molecule
EpCAM^high^ CTC: EpCAM high expressing CTC
EpCAM^low^ CTC: EpCAM low expressing CTC
mBC: metastatic breast cancer
