# Supplementary material for: EpCAMhigh and EpCAMlow circulating tumor cells in metastatic prostate and breast cancer patients
Source: Oncotarget. 2018 Nov 2;9(86):35705–16. doi: 10.18632/oncotarget.26298 (PMC6235023; doi:10.18632/oncotarget.26298)
Supplement: Supplementary file 1 [file oncotarget-09-35705-s001.pdf]

EpCAM^{high} and EpCAM^{low} circulating tumor cells in metastatic prostate and breast cancer patients

SUPPLEMENTARY MATERIALS

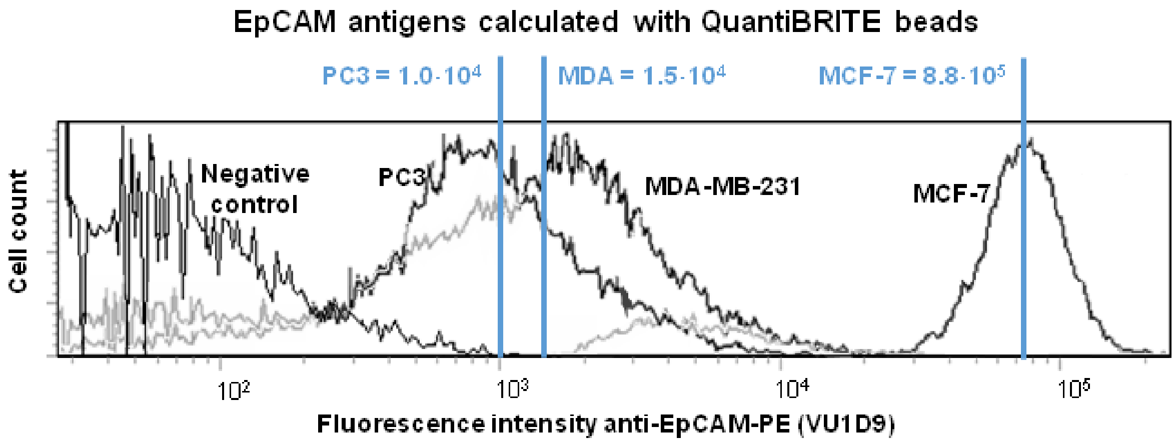

Supplementary Figure 1: Distribution of EpCAM density on cancer cell lines. Histograms showing the distribution of EpCAM density of cancer cell line PC3, MDA-MB-231 and MCF-7 cells, as well as a negative control. The actual number of antigens (blue) was determined by the use of QuantiBRITE beads (BD Bioscience). The average EpCAM density of PC3 is 1.0×10^4 antigens with 89% CV. The average EpCAM density of MDA-MB-231 is 1.5×10^4 antigens with a CV of 165%. The average EpCAM density of MCF-7 is 8.8×10^5 antigens with a CV of 62%.

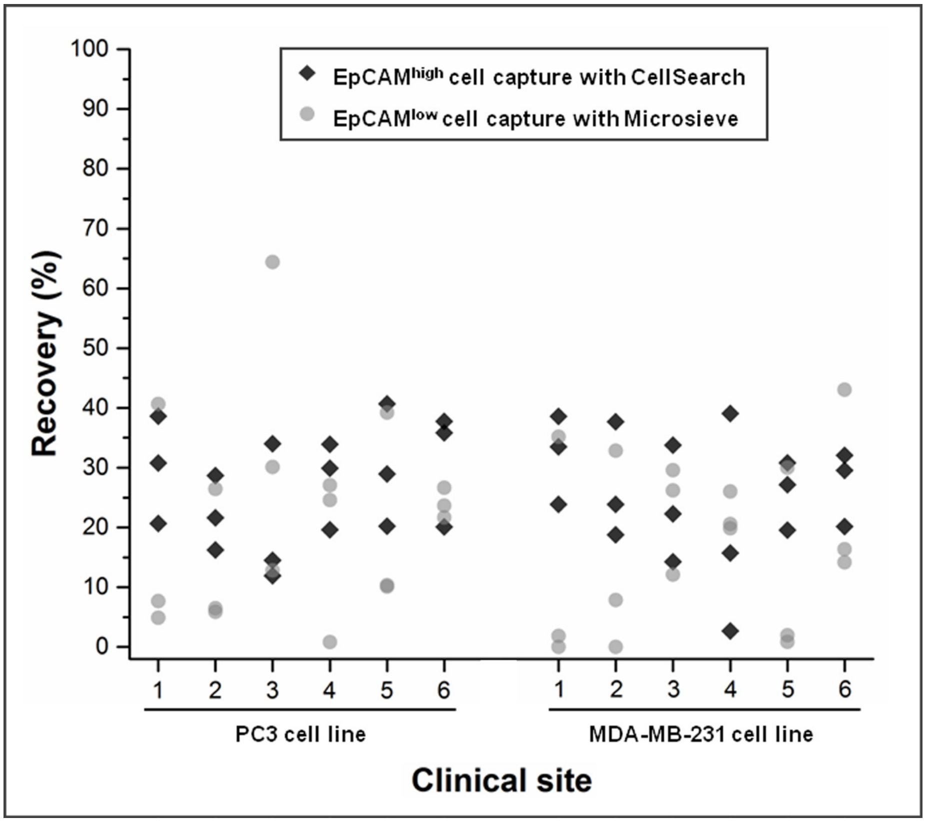

Supplementary Figure 2: Recovery of PC3 and MDA-MB-231 cells per clinical site. Scatter plot displaying the recovery (%) of PC3 and MDA-MB-231 cells of each processed sample with CellSearch and microsieves per clinical site.

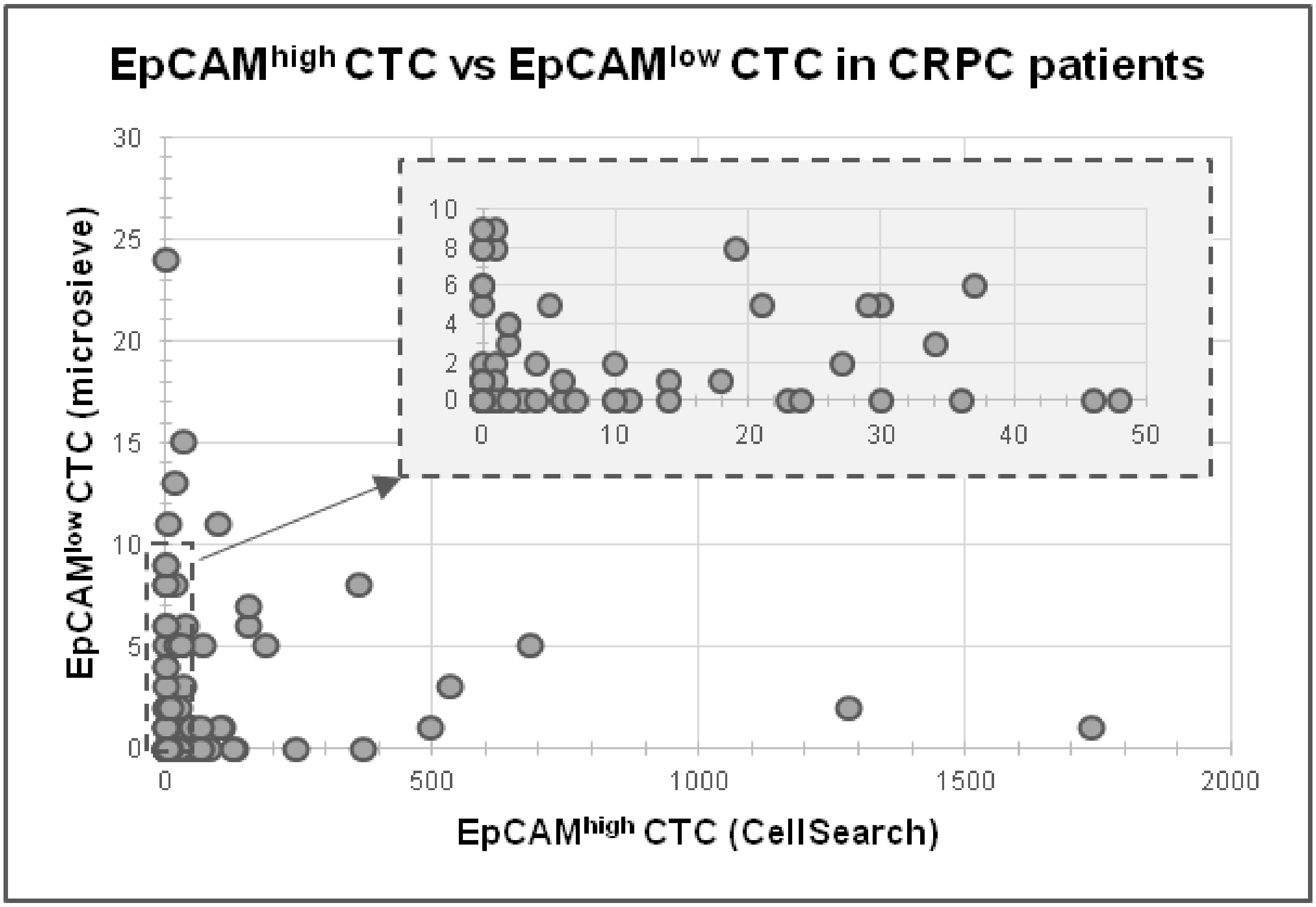

Supplementary Figure 3: CTC counts in 91 CRPC patients. Scatter plot of $EpCAM^{high}$ and $EpCAM^{low}$ CTC counts in 91 castration-resistant prostate cancer patients.

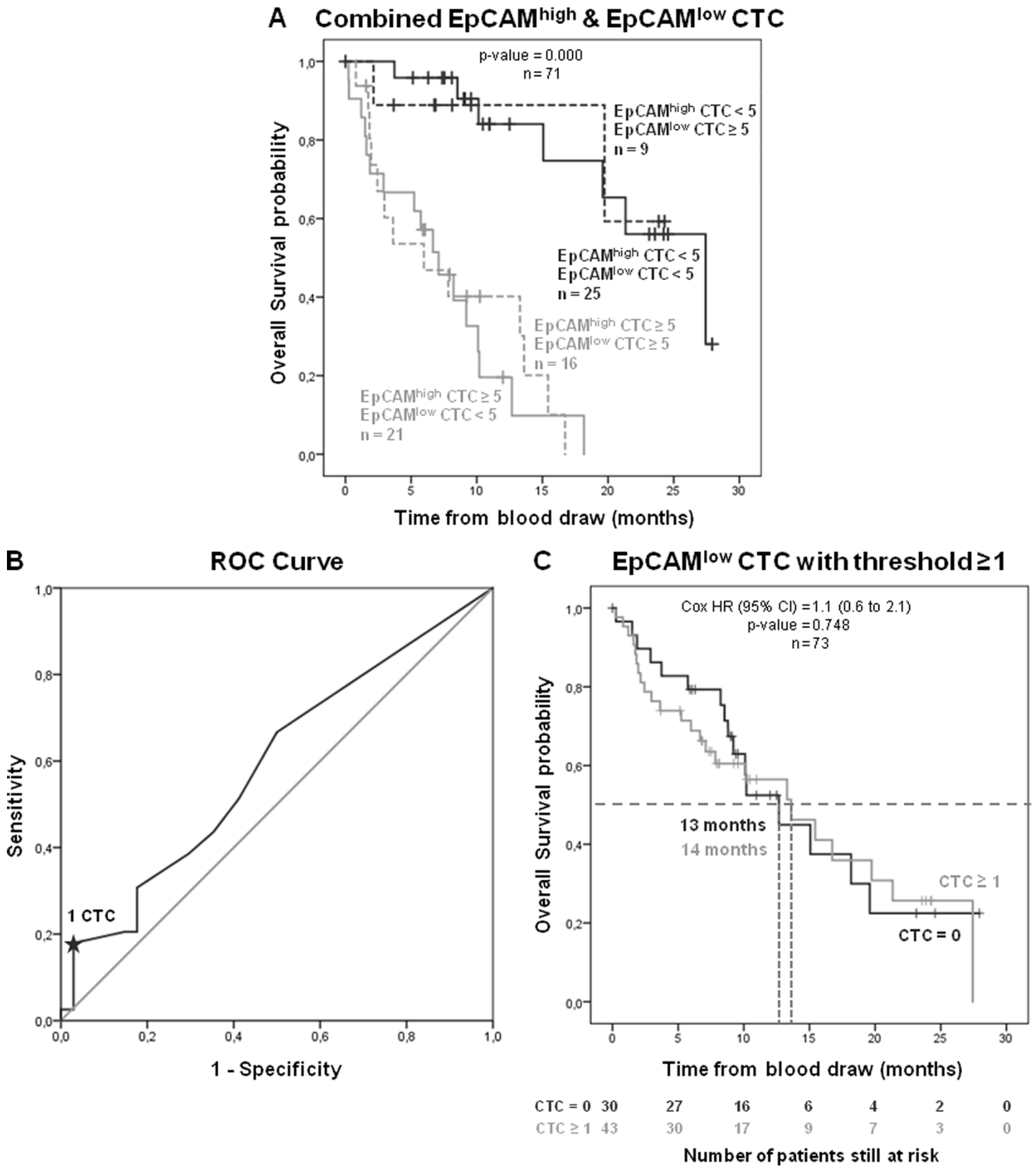

Supplementary Figure 4: Overall survival for EpCAMlow CTC. Kaplan–Meier curves of 71 CRPC patients separated into four groups with both EpCAMhigh and EpCAMlow CTC measured (**A**): 1. <5 EpCAMhigh CTC and <5 EpCAMlow CTC (black solid line); 2. <5 EpCAMhigh CTC and ≥5 EpCAMlow CTC (black dashed line); 3. ≥5 EpCAMhigh CTC and <5 EpCAMlow CTC (grey solid line); and 4. ≥5 EpCAMhigh CTC and ≥5 EpCAMlow CTC (grey dashed line). It shows a strong trend between EpCAMhigh CTC and survival, whereas the presence or absence of EpCAMlow CTC has no influence on survival. The ROC curve indicated that the highest diagnostic ability for EpCAMlow CTC is with a threshold of 1 CTC (**B**). The Kaplan-Meier curve for overall survival of EpCAMlow CTC with 1 CTC threshold shows no correlation with survival ($p = 0.748$) (**C**).

Supplementary Table 1: Overview of CTC found in all CRPC and mBC patients. Detailed overview of EpCAMhigh and EpCAMlow CTC found in castrate-resistant prostate cancer patients and metastatic breast cancer patients. See Supplementary_Table_1
